# Racial Disparities and the Effect of County Level Income on the Incidence and Survival of Young Men with Anal Cancer

**DOI:** 10.1089/heq.2018.0018

**Published:** 2018-08-01

**Authors:** Markian M. Bojko, Robert J. Kucejko, Juan L. Poggio

**Affiliations:** ^1^ Department of Surgery, Drexel University College of Medicine, Philadelphia, Pennsylvania.; ^2^ Division of Colorectal Surgery, Department of Surgery, Drexel University College of Medicine, Philadelphia, Pennsylvania.

**Keywords:** anal cancer, incidence and survival, racial disparity, socioeconomic status

## Abstract

**Purpose:** Prior studies have identified a racial disparity in incidence and survival of squamous cell carcinoma of the anus (SCCA) in the young African American male population. We aim to determine whether racial disparities are independent of income and urban location.

**Methods:** The National Cancer Institute's Surveillance of Epidemiology and End Results database was queried for data on patients with SCCA for the years of 2000–2013. Cox regression was used to determine the effect of race, county median family income, rural–urban continuum, and stage of disease on overall survival.

**Results:** The incidence rate of SCCA was significantly higher in black men <50 years old than in white men. Black race had a hazard ratio of 1.55 (confidence interval [CI] 1.33–1.81) when controlling for age, stage, income, and urban–rural status. Each $10,000 increase in county median family income was protective with a hazard ratio of 0.90 (CI 0.86–0.94). Residence in a metropolitan area did not significantly affect survival.

**Conclusions:** The lower survival of black men <50 years old with SCCA is independent of income, urban location, and stage of disease. Further efforts are needed to target this at-risk population and the authors suggest wide application of previously validated screening programs for anal dysplasia.

## Introduction

The incidence rates of anal cancer have been increasing in the United States significantly over the past two decades. The relative risk (RR) for anal cancer in men aged 20–34 years was four times as high as to women of the same age between 1997 and 2009.^1,2^ The average annual percentage change in this same age group was 10.7% in men, which was more than double the rate in women at 4.0%. Similarly, the RR for anal cancer in men 35–49 years was almost two and a half times higher that of women. The reason for this disproportionate incidence of anal cancer in these age groups is not well understood in the literature. The unequal incidence is not seen in older patients. These reports demonstrate that young men have emerged as the most vulnerable demographic subgroup at risk for developing anal cancer.

Prior research suggests that the increased incidence seen in the young male population is due to changing sexual practices.^1^ Approximately 85% of the anal cancer cases are squamous cell carcinoma of the anus (SCCA): a disease believed to be etiologically associated with human papillomavirus (HPV) infection. The risk factors for contraction of HPV include lifetime number of sexual partners, sexual practices, race, age, same-sex sexual behavior, and underlying human immunodeficiency virus (HIV) infection. Thus, among the highest risk for development of anal cancer are HIV positive men who have sex with men (MSM), showing a RR of 59.5.^3^ Although many individuals who are infected with HPV spontaneously clear the virus without showing any symptoms, those infected with HIV are at increased risk of persistent HPV infection and, therefore, at increased risk for anal cancer.^4^ The most recent report by the CDC stated an HIV incidence rate of 44.3 (per 100,000) for African Americans compared with 5.3 for Caucasians. Furthermore, 70% of new infections were attributed to male-to-male sexual intercourse and injection drug use.^5^ Moreover, between 2000 and 2011, the RR for anal cancer in black patients <30 years old was reported to be 4.56 when compared with non-Hispanic white patients of similar age.^6^

Black race has also been consistently reported as a poor prognostic factor for the survival of anal cancer patients. Between 1973 and 2000, 5-year relative survival in black men decreased from 45% to 27%, whereas survival rates increased for white men and women.^2^ Multivariate analysis of anal cancer cases between 1973 and 2012 identified black race as being an independent predictor of lower survival.^7,8^ Yet, the effect of socioeconomic status was not evaluated in these studies. The rising incidence of SCCA in the young male population, coupled with decreased survival in black men, raises significant concern for this population. It is necessary to identify any associated social factors that could be responsible for this racial disparity, to optimize outreach efforts. The aim of this study is to determine the effect of socioeconomic variables such as median family income and rural–urban continuum on incidence and survival in young men, as they are disproportionately at risk for SCCA. Further understanding of these variables will allow for better delineation of those most at risk, allowing for improved medical education outreach. Data from the Surveillance, Epidemiology and End Results (SEER) database will be analyzed to address this hypothesis.

## Methods

After exemption from institutional review board approval was obtained, the SEER data set was queried for male patients, 18–49 years old, with histologically confirmed SCCA as their only cancer diagnosis between the years of 2000 and 2013, chosen to best correspond to census data on median family income. Cases were excluded if race was unknown, disease was unstaged, microscopic confirmation was not present, or if they were not actively followed in the database. Anal cancer was defined using the International Classification of Diseases for Oncology version 3 (ICD-0-3) site codes C21.0, C21.1, and C21.8. Squamous cell carcinoma was defined by SEER histology codes 8070–8078. Stage was defined using SEER definitions of *in situ*, localized, regional, and distant disease.

Incidence rates were standardized to the 2000 U.S. standard population. RR was calculated using incidence rate ratios. Cumulative expected survival rates were calculated using the Ederer II method, and relative age standardized survival rates were calculated using the actuarial method. Stage was evaluated according to the SEER-defined staging variable: summary stage 2000 (1998+). Median family income was determined on a county attribute level, based on data obtained from the Census American Community Survey 5-year files. Rural–urban category was determined using the United States Department of Agriculture Rural-Urban continuum codes. SEER*Stat version 8.3.4 and SPSS version 24.0.0.0 were used for Mann–Whitney *U* tests, Cox Proportional Hazards Survival functions using 95% confidence intervals (CI), and *p* values of 0.05 to determine statistical significance.

## Results

A total of 28,420 cases of cancer located in the anus, anal canal, and anorectum between 2000 and 2013 were identified in the SEER database. Of these, 21,425 were microscopically confirmed squamous cell carcinoma according to histology codes and 10,963 cases were men. Of these men, 5209 were <50 years old, and after excluding cases of unknown race and stage, 4679 men were identified with SCCA between 2000 and 2013. In the final sample, 78.5% of patients were white, 18.0% were black, and 65.5% of cases were men aged 40–49 years. The incidence in black men aged 35–49 years was 4.393 compared with 3.136 in white men (*p*<0.001), representing a rate ratio of 1.401. The incidence in black men <35 years was 0.505 compared with 0.246 in white men (*p*<0.001), representing a rate ratio of 2.052. On presentation, black men had more advanced disease than white men. Incidence rates by stage and rate ratios with respect to race are presented in Table 1. The incidence of *in situ* disease in patients residing in metropolitan areas was nearly four times higher than that of any other stage or residential location (Table 2).

**Table 1.** **Incidence Rates and Rate Ratios of Squamous Cell Carcinoma of the Anus by Race, Stratified by Stage, in White and Black Males <50 Years Old, 2000–2013**

	Black		White			
Stage	*N*	Rate	*N*	Rate	B:W rate Ratio	*p*
*In situ*	471	0.978	2564	0.827	1.18	0.001
Localized	197	0.422	696	0.224	1.89	<0.001
Regional	137	0.293	334	0.107	2.73	<0.001
Distant	35	0.073	81	0.026	2.80	<0.001

Rates are per 100,000 and age adjusted to the 2000 U.S. standard population.

**Table 2.** **Incidence Rates of Squamous Cell Carcinoma of the Anus by Rural–Urban Continuum Stratified by Stage, in Males <49 Years Old, 2000–2013**

Rural–urban category	Stage	*N*	Rate	Confidence interval
Metropolitan counties	*In situ*	3090	0.843	0.813–0.873
	Localized	845	0.232	0.217–0.249
	Regional	458	0.126	0.114–0.138
	Distant	109	0.029	0.024–0.035
Non-Metropolitan counties	*In situ*	74	0.203	0.160–0.255
	Localized	66	0.174	0.135–0.222
	Regional	25	0.067	0.044–0.100
	Distant	12	0.031	0.016–0.055

Rates are per 100,000 and age-adjusted to the 2000 U.S. standard population.

**FIG. 1.**
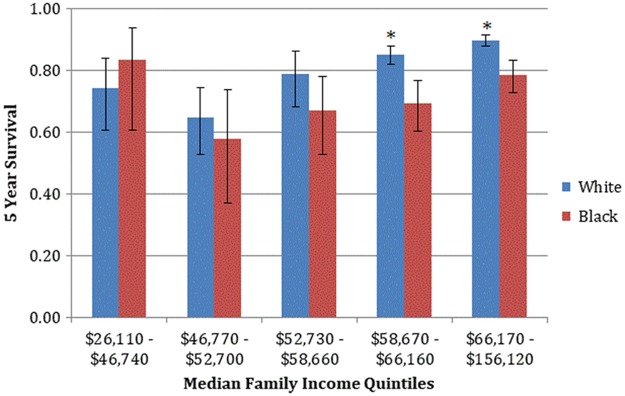
Five-year age-standardized survival for males <49 years old, with squamous cell carcinoma of the anus, 2000–2013, by median family income (* denotes a statistically significant difference between white and black confidence intervals).

The 5-year relative age standardized survival was significantly lower for black men <50 years old (0.738, CI 0.696–0.776) than for white men (0.870, CI 0.855–0.884). Although there is a general decrease in survival with more progressive stages of disease, a significant racial disparity was identified among patients with *in situ* and localized disease. White men had significantly higher 5-year survival with *in situ* (0.954 vs. 0.892, *p*<0.05) and localized disease (0.791 vs. 0.628, *p*<0.05) than black men. White men in the highest two income quintiles had significantly improved 5-year survival across all stages than black men (Fig. 1).

**FIG. 2.**
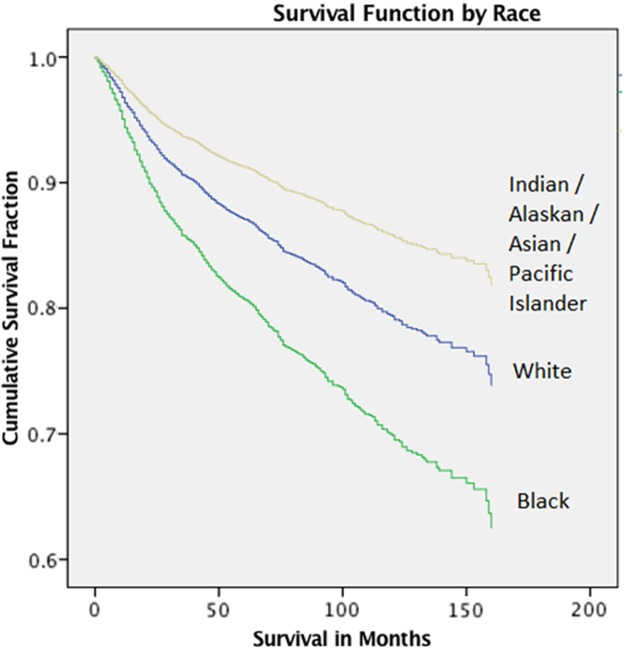
Cox regression survival function for race, adjusted for stage, and median family income.

Based on the Cox Proportional Hazards model, black race was a poor prognostic factor for survival independent of stage, age, median family income, and rural–urban category (Table 3). Higher median family income was protective for survival, with each increase in median family income of $10,000 associated with a 10% benefit to overall survival. Advanced stage significantly affected survival with localized, regional, and distant stages having significantly lower overall survival than *in situ* disease. Age and metropolitan residence did not significantly affect overall survival. The survival function curve based on the Cox regression model illustrates the significantly lower survival associated with black race when adjusted for median family income and stage (Fig. 2).

**Table 3.** **Cox Regression Model for Determinants of Survival in Male Patients <50 Years Old with Squamous Cell Carcinoma of the Anus**

	Hazard ratio	Confidence interval
Race		
White (ref)	1	—
Black	1.556	1.332–1.817
Stage		
*In situ*	1	—
Localized	2.901	2.438–3.454
Regional	5.572	4.648–6.678
Distant	14.436	11.182–18.636
Median family income^a^	0.901	0.862–0.943
Age	1.056	0.993–1.123
Rural–urban^b^	1.061	0.789–1.426

^a^ Median family income variable was defined by increments of $10,000.

^b^ Rural–urban category compares hazard ratio of cases in nonmetropolitan counties with those in metropolitan counties.

## Discussion

The data demonstrate increased incidence of later stage disease in SCCA among black men than among white men, with nearly three times as many black men diagnosed with regional and distant disease at the time of presentation. Differences in the prevalence of HIV infection with respect to race is likely a major contributor to the racial disparity seen in the incidence of SCCA.^5^ Prior research indicates that elevated HIV rates among black MSM can be explained by high rates of undiagnosed seropositivity, high rates of sexually transmitted infections, low use of antiretroviral therapy (ART), and low adherence to ART.^9^ These factors likely apply to the incidence patterns observed here in SCCA. The CDC identifies inadequate HIV prevention education and interventions, limited awareness of infection, low perception of risk, alcohol and illegal drug use, and feelings of rejection and isolation as factors that may be contributing to the increased burden of HIV infection in this population.^10^ As HIV and SCCA are intimately related, addressing these factors through culturally sensitive education would likely decrease SCCA incidence.

We observed significantly higher incidence of overall disease, as well as a large shift toward diagnosis at earlier stages in metropolitan counties than nonmetropolitan counties. Similarly, McKenney et al. have shown decreased incidence of HIV in MSM in rural areas but worse outcomes. They attribute this to the lack of culturally appropriate outreach and prevention.^11^ A recent study of 1907 men (901 HIV+) from four major metropolitan areas identified a number of factors significantly associated with increased intention to seek screening. These factors included any history of anal condyloma, awareness of available screening in the community, current possession of health insurance, or current HIV infection.^12^ Studies specifically evaluating the barriers to screening for anal cancer among MSM cite unique barriers such as a lack of awareness of the disease in the patient, as well as a lack of healthcare recommendations to screen. They suggest an emphasis on healthcare literacy among at-risk groups to empower them to prevent cancer, more training for healthcare professionals, specifically those who routinely treat at-risk patients, and the development of national guidelines to promote anal dysplasia screening.^13^

Improved education surrounding the use of the HPV vaccination would also serve to decrease incidence of SCCA. The HPV vaccine is recommended for all men up to age 21 years, and through age 26 years for MSM. Yet, actual vaccination rates are dismal ranging from 4.9% to 21% in ages 18–26 years. A key barrier to vaccination is the stigma surrounding asking for the vaccine, as youths feel it may identify them as promiscuous.^14^ Providers need to proactively address possible stigma to better educate their patients on the benefits of vaccination. This would serve to improve vaccination rates, thereby decreasing incidence of SCCA.

Yet, incidence is only half of the battle. The significant racial disparity in survival between young men in this study was independent of county level income and stage of diagnosis. After adjustment for stage and median family income, black men continued to have lower survival than white men. Although many studies have identified treatment interventions that work in black MSM with HIV, very few have analyzed why outreach to black MSM with HIV tends to fall short.^15^ It is reasonable to assume that similar limitations apply when treating SCCA in black MSM, which would explain the lower observed survival seen in the regression analysis. Willeford et al. have noted significantly less follow-up with young black men, which is associated with higher rates of high-grade anal dysplasia.^16^ Failure to follow up with SCCA would also lead to lower survival.

Structural barriers, including stigma, racism, incarceration, and other barriers to care, have a significant impact on black men seeking healthcare.^17^ These barriers have been studied extensively in the realm of HIV treatment, and the conclusions are directly attributable to the health disparity in SCCA. Addressing the stigma surrounding MSM and anal health, in general, would serve to improve survival. Many young men feel stigmatized about their sexual practices, and anal health, in general, which lends to avoidance of the healthcare system.^18^ Community outreach programs would lead to earlier diagnosis and improved survival in SCCA, as was seen in the San Francisco SEER registry after implementation of new screening programs.^5^ Further research on the comparative effectiveness of culturally specific screening and health educational programs with respect to anal health would further improve outcomes in SCCA.

The limitations of this study include the inability to determine HIV status of individual patients within the SEER database, therefore, the effect of HIV prevalence on incidence and survival cannot be analyzed. Second, insurance status is not available in the SEER database, which may affect the likelihood of a patient to seek medical care. Third, income and geographic location are generalized to counties, and do not equally affect all patients living within that county. This limits the direct implication of county-level income on individual mortality. However, county income inequality is a significant determinant of health and controlling for it in regression analysis lends power to other factors such as race, and allows for the development of health improvement strategies that are applicable across the United States.^19^

## Conclusions

Black race remains a poor prognostic indicator of survival of SCCA, even after controlling for median family income and stage. The health inequity identified here for diagnosis and treatment of SCCA likely parallels that studied for HIV, and using similar approaches to address this would greatly improve incidence and survival in SCCA.

Improved screening protocols would identify SCCA at earlier stages and improve overall survival in at-risk populations. We advocate for an expansion of screening protocols across the United States, with an emphasis on at-risk populations across all races. But, special emphasis should be placed on developing culturally sensitive programs designed for young black men, as this was shown to be a poor prognostic indicator. Patients will further benefit from proactive physicians who openly talk about anal health, HPV vaccination, and regular screening to create a safe space in their clinics for all patients.

## Abbreviations Used

ART: antiretroviral therapy
CI: confidence interval
HIV: human immunodeficiency virus
HPV: human papillomavirus
RR: relative risk
SCCA: squamous cell carcinoma of the anus
SEER: surveillance, epidemiology, and end results

## References

[1] Nelson RA, Levine AM, Bernstein L, et al. Changing patterns of anal canal carcinoma in the United States. J Clin Oncol. 2013;31:1569–1575 23509304 10.1200/JCO.2012.45.2524 PMC3753461

[2] Johnson LG, Madeleine MM, Newcomer LM, et al. Anal cancer incidence and survival: the surveillance, epidemiology, and end results experience, 1973–2000. Cancer. 2004;101:281–288 15241824 10.1002/cncr.20364

[3] Abbas A, Yang G, Fakih M Management of anal cancer in 2010. Part 1: overview, screening, and diagnosis. Oncology (Williston Park). 2010;24:364–369 20464850

[4] van de Laar TJ, Richel O Emerging viral STIs among HIV-positive men who have sex with men: the era of hepatitis C virus and human papillomavirus. Sex Transm Infect. 2017;93:368–373 27789574 10.1136/sextrans-2016-052677

[5] Amirian ES, Fickey PA Jr, Scheurer ME, et al. Anal cancer incidence and survival: comparing the greater San-Francisco bay area to other SEER cancer registries. PLoS One. 2013;8:e58919 23484057 10.1371/journal.pone.0058919 PMC3590168

[6] Shiels MS, Kreimer AR, Coghill AE, et al. Anal cancer incidence in the United States, 1977–2011: distinct patterns by histology and behavior. Cancer Epidemiol Biomarkers Prev. 2015;24:1548–1556 26224796 10.1158/1055-9965.EPI-15-0044 PMC4592448

[7] Kim E, Kim JS, Choi M, et al. Conditional Survival in Anal Carcinoma Using the National Population-Based Survey of Epidemiology and End Results Database (1988–2012). Dis Colon Rect. 2016;59:291–298 10.1097/DCR.0000000000000555 26953987

[8] Metildi C, McLemore EC, Tran T, et al. Incidence and survival patterns of rare anal canal neoplasms using the surveillance epidemiology and end results registry. Am Surg. 2013;79:1068–1074 24160801 PMC4209843

[9] Oster AM, Wiegand RE, Sionean C, et al. Understanding disparities in HIV infection between black and white MSM in the United States. AIDS (London, England) 2011;25:1103–1112 10.1097/QAD.0b013e3283471efa 21505305

[10] Centers for Disease Control and Prevention. HIV Surveillance Report, 2015 Vol. 27 Available at www.cdc.gov/hiv/library/reports/hiv-surveillance.html 2016. Accessed 15, 2017

[11] McKenney J, Sullivan PS, Bowles KE, et al. HIV risk behaviors and utilization of prevention services, urban and rural men who have sex with men in the United States: results from a National Online Survey. AIDS Behav. 2017;22:2127–2136 10.1007/s10461-017-1912-5 28986669

[12] D'Souza G, Cook RL, Ostrow D, et al. Anal cancer screening behaviors and intentions in men who have sex with men. J Gen Intern Med. 2008;23:1452–1457 18618198 10.1007/s11606-008-0698-6 PMC2518019

[13] Koskan AM, LeBlanc N, Rosa-Cunha I Exploring the perceptions of anal cancer screening and behaviors among gay and bisexual men infected with HIV. Cancer Control. 2016;23:52–58 27009457 10.1177/107327481602300109 PMC4928714

[14] Fontenot HB, Fantasia HC, Vetters R, et al. Increasing HPV vaccination and eliminating barriers: recommendations from young men who have sex with men. Vaccine. 2016;34:6209–6216 27838067 10.1016/j.vaccine.2016.10.075

[15] Maulsby C, Millett G, Lindsey K, et al. A systematic review of HIV interventions for black men who have sex with men (MSM). BMC Public Health. 2013;13:625 23819660 10.1186/1471-2458-13-625 PMC3710496

[16] Willeford WG, Barroso L, Keller J, et al. Anal dysplasia screening and treatment in a southern human immunodeficiency virus clinic. Sex Transm Dis. 2016;43:479–482 27419814 10.1097/OLQ.0000000000000475 PMC5872814

[17] Levy ME, Wilton L, Phillips G II, et al. Understanding structural barriers to accessing HIV testing and prevention services among black men who have sex with men (BMSM) in the United States. AIDS Behav. 2014;18:972–996 24531769 10.1007/s10461-014-0719-x PMC4509742

[18] Radcliffe J, Doty N, Hawkins LA, et al. Stigma and sexual health risk in HIV-positive African American young men who have sex with men. AIDS Patient Care STDs. 2010;24:493–499 20673080 10.1089/apc.2010.0020 PMC4932787

[19] Lopez DB, Loehrer AP, Chang DC Impact of income inequality on the Nation's Health. J Am Coll Surg. 2016;223:587–594 27457253 10.1016/j.jamcollsurg.2016.07.005

